# Disentangling visually driven tactile predictions from multisensory integration in body ownership

**DOI:** 10.1098/rsos.252132

**Published:** 2026-04-01

**Authors:** Renzo C. Lanfranco, Marie Chancel, Birgit Hasenack, H. Henrik Ehrsson

**Affiliations:** ^1^ Department of Clinical Neuroscience, Karolinska Institutet, Stockholm, Sweden; ^2^ Department of Neuroscience, Karolinska Institutet, Stockholm, Sweden; ^3^ Centre de Recherche en Psychologie et Neurosciences, Aix-Marseille Université, Marseille, France; ^4^ Faculty of Social and Behavioural Sciences, Utrecht University, Utrecht, The Netherlands

**Keywords:** body ownership, multisensory perception, predictive processing, psychophysics, rubber hand illusion

## Abstract

The sense of owning one’s body is essential for distinguishing internal from external sensations. Research using bodily illusions shows that this sense depends on integrating visual, tactile and proprioceptive signals, yet the role of multisensory predictions remains unclear. We addressed this using a modified rubber hand illusion. Two robotic arms tapped the two visible rubber hands, while a third arm simultaneously tapped the participant’s hidden real hand. One rubber hand received synchronous stimulation, while the other was stimulated with varying temporal delays. Visual feedback was manipulated across three conditions: full view of the approaching probe and touch, occlusion of the approaching probe or occlusion of the touch event. Participants reported which rubber hand felt more like their own hand. Using signal detection analysis, we examined whether occluding predictive visual feedback influenced sensitivity to visuotactile synchrony, perceptual bias or both. Results showed that occluding the approaching probe did not reduce sensitivity to visuotactile signals; as long as the touch or its timing was visible or inferable, sensitivity remained stable. However, perceptual bias was reduced when predictive visual information was removed. These findings reveal how visually driven tactile predictions shape body ownership and highlight signal detection analysis as a useful tool to dissociate sensitivity and bias in self-perception.

## 1. Introduction

The sense that one’s body belongs to oneself plays a crucial role in distinguishing between internal and external sources of sensory information [1,2]. This sense of body ownership arises from the brain’s integration of visual, tactile, proprioceptive and other bodily signals, following principles of temporal and spatial congruence in multisensory integration [1,3] and perceptual inference that these signals originate from a common cause [4]. Developing a sense of bodily self equips us with more effective means to perceive and protect our bodies by supporting predictions about how external events will affect the body [5,6]. However, the specific role of sensory predictions in shaping the sense of body ownership—and their underlying perceptual processes and neural mechanisms—remains poorly understood.

A substantial body of research demonstrates that the capacity to generate predictions is fundamentally linked to both perception and action. Sensory predictions, in particular, are critical for shaping anticipatory motor behaviours [7–12], enabling the organism to anticipate potential threats via top-down modulatory mechanisms [13,14]. One example is how visual predictions can influence perception in other sensory modalities, especially touch. For example, when a looming visual stimulus aligns spatially and temporally with the predicted impact of a tactile event, tactile detection is significantly enhanced [15,16]. These findings raise the question of whether similar predictive mechanisms operate in body ownership, where the brain must continuously predict and evaluate multisensory consequences of events affecting the body.

Evidence from neurophysiology suggests a potential neural basis for such multisensory predictions. Hyvärinen & Poranen [17] recorded neurons in area 7b of the inferior posterior parietal cortex with tactile receptive fields (e.g. on the arm or face) that also responded to visual stimuli approaching those same body parts [17]. They interpreted these visual responses as ‘anticipatory activation’, since the neurons increased their firing before any tactile contact occurred. Further evidence comes from Duhamel *et al.* [18], who found that most neurons in the ventral intraparietal area (VIP) are bimodal [18], responding to both tactile and visual stimuli with overlapping receptive fields. Many of these VIP neurons are also motion-sensitive, particularly to ‘looming’ stimuli approaching the monkey, with little or no response to stimuli moving away. Avillac *et al.* [19] demonstrated that these neurons encode stimulus location in a body part-centred reference frame, further supporting the idea that they represent space relative to the body rather than purely in retinotopic coordinates [19]. Given that multisensory neurons implicated in body ownership and multisensory neurons that show predictive responses to approaching stimuli have both been described, it is plausible that predictive mechanisms contribute to the experience of owning one’s body.

To investigate body ownership in experimental settings, researchers often employ bodily illusions such as the rubber hand illusion (RHI) [20]. In the traditional RHI paradigm, both the participant’s hidden real hand and a visible rubber hand are stroked in synchrony and at a constant rhythm. This coordinated multisensory stimulation gives rise to the compelling experience that the rubber hand is part of one’s own body, and that tactile and proprioceptive sensations from the real hand are perceived as originating from the rubber hand [21]. The intensity of this ownership illusion is usually measured through subjective questionnaires [20,22,23], proprioceptive drift (a perceived shift in hand position towards the rubber hand [20,24–26]), and more recently, objective two-alternative forced-choice (2AFC) psychophysical paradigms [27–32].

In the psychophysical variant of the RHI, two rubber hands are presented at once, each receiving visuotactile stimulation that varies in its temporal synchrony with the participant’s real hand. Participants indicate which of the two rubber hands (left or right) feels more like their own. The proportion of trials in which ownership is attributed to each hand is then computed for every level of visuotactile asynchrony. These data can be examined by fitting psychometric curves [27,28] or by applying signal detection theory (SDT) to assess sensitivity to multisensory cues underlying body ownership [29–33]. The 2AFC method thus enables estimation of perceptual discrimination thresholds between the concurrently induced RHIs.

Although psychometric function fitting and SDT can both be applied to 2AFC data, they differ in the level at which sensitivity is defined. In psychometric analyses, sensitivity is typically inferred from the slope of the psychometric function or from the ‘just noticeable difference’ (JND), which reflects the scale of internal variability governing changes in choice probability across stimulus values. However, JNDs are inherently procedure dependent and can vary with stimulus spacing, uncertainty and response format, even when underlying sensory discriminability remains constant [34–36]. In contrast, SDT defines sensitivity as *d′*, which quantifies the separation between internal evidence distributions associated with the two sensory alternatives, measured relative to their variance. This formulation yields a task general measure of perceptual discriminability that is directly comparable across paradigms, including 2AFC tasks, and is explicitly constructed, under standard SDT assumptions, to be independent of decision criteria. Because the present study aims to test whether disrupting visually driven tactile predictions alters the *precision* with which visuotactile synchrony is discriminated by body ownership, rather than merely shifting choice preferences, an SDT-based analysis using *d*′ provides a more appropriate metric of sensitivity than JND.

SDT has traditionally been used to measure both sensitivity, which corresponds to the ability to distinguish signal from noise, and the decision criterion, which represents the threshold or tendency to report the presence of a signal [35,37]. The decision criterion has often been interpreted as reflecting post-perceptual internal biases. However, research on perceptual illusions has shown that this measure can capture not only response bias but also perceptual bias. Importantly, perceptual bias reflects fundamental perceptual features of the illusion under investigation [38–42]. In 2AFC tasks, which are largely resistant to response bias [35,43–49], the decision criterion primarily reflects perceptual biases produced by the illusion itself. Recent findings from our group demonstrate that SDT provides a useful framework for quantifying how multisensory factors influence body ownership in the RHI. Specifically, visuotactile asynchrony modulates ownership sensitivity [29–31], spatial distance between the real and rubber hand affects both sensitivity and perceptual bias [30], and texture congruence selectively influences perceptual bias [31]. Together, these results illustrate how SDT can disentangle distinct sensory and perceptual components that underlie the illusion.

Previous behavioural research on the RHI has primarily focused on the role of multisensory integration in body ownership, but a few studies have begun to directly investigate the contribution of sensory predictions. Ferri *et al.* [50] reported that merely observing a visual stimulus slowly approaching the rubber hand—without any actual touch—was sufficient to elicit a sense of ownership over that limb [50]. In their experiment, the experimenter’s hand moved towards the rubber hand at 2 cm s^−1^ from a distance of 70 cm, stopping 15 cm above it, with no tactile stimulation delivered to the real hand. This slow approach, which terminates well before contact, is unlikely to elicit precise tactile predictions, and the absence of actual tactile input prevents such predictions from being matched or integrated with somatosensory signals. A conceptual replication failed to reproduce the effect, concluding instead that the RHI is primarily driven by bottom-up integration of visual and somatosensory signals [51]. However, it may still be the case that the match between visually driven tactile predictions, generated when an object moves towards and contacts the hand, and actual tactile feedback from the corresponding part of the hand at the expected time can trigger the RHI. In line with this idea, Heurley *et al.* [52] showed that synchronous brushing of the rubber and real hands, even when the final part of the brush trajectory contacting the rubber hand was occluded, produced a similarly strong illusion as in the classical condition without occlusion [52]. However, these studies all relied on questionnaire ratings [50–52] and, in some cases, skin conductance responses [50,51], precluding the assessment of more fine-grained changes in psychometric functions or perceptual discrimination thresholds afforded by psychophysical approaches.

Using a 2AFC task, Chancel *et al.* [27] investigated whether occluding the first two-thirds of the visual trajectory of a robotic arm approaching and touching a rubber hand, thereby preventing visually driven tactile predictions, would influence the sense of hand ownership [27]. In their study, they selectively occluded either the approach of the robotic arm towards the rubber hand, the moment of contact between the robotic arm and the rubber hand, or presented the full trajectory without occlusion. They measured participants’ points of subjective equality (PSE) across different levels of visuotactile asynchrony. The PSE served as an indicator of perceptual bias. Specifically, participants’ preference for the sensation of ownership over one rubber hand versus the other, as induced by the RHI. Their results showed a significant shift in PSE when the visibility of the robot arm’s approach was occluded, indicating a lower proportion of ownership judgements compared to conditions where only the contact event was occluded or where the full visual trajectory was visible. This finding suggests that body ownership depends more on visually driven tactile predictions than on the sight of the touch event, as participants reported a higher proportion of ownership judgements when visual information supporting tactile prediction was available. These results indicate that tactile visually driven tactile predictions influence the body ownership experience.

There are, however, several questions that remain open. Does occluding the approach of the object merely shift perceptual bias or does it also reduce body ownership sensitivity to visuotactile synchrony? Conversely, when the object’s approach is visible but the final contact is hidden, can the visual system extrapolate unseen motion behind the occluder precisely enough to allow an effective match between the tactile signals and the expected point of contact, or is body ownership sensitivity to visuotactile asynchrony affected? The traditional psychometric analysis used by Chancel *et al.* [27] could not separate perceptual bias from sensitivity and could therefore not directly address these questions, leaving it unclear as to which process underlies the observed effects [27].

In the present study, we applied an SDT framework to reanalyse the dataset from Chancel *et al.* [27] in order to separate two components of body ownership: sensitivity to visuotactile synchrony and perceptual bias linked to the illusion [30,31]. This approach enabled us to examine whether disrupting visually driven tactile predictions influences the precision of visuotactile integration, the perceptual bias underlying ownership judgements or both. Specifically, we investigated whether occluding the approach of the robotic arm, and thereby preventing tactile predictions, decreases body ownership sensitivity to visuotactile synchrony or merely shifts the perceptual bias of the RHI. We also assessed whether body ownership sensitivity remains unchanged when the approach is visible but the final touch event is occluded, which would suggest that the visual system can extrapolate unseen motion and generate tactile predictions precise enough to substitute the actual visual feedback of touch with an inferred moment of contact.

## 2. Method

### 2.1. Participants

We conducted an *a priori* power analysis using the pwrss R package (v. 0.3.1) on pre-existing datasets that employed the same 2AFC detection paradigm [28,30]. This analysis determined that a sample size of 20 participants was necessary to achieve a power of 0.8, when aiming at an effect size of $\eta p^{2}=0.4$ and an alpha of 0.05. Consequently, we recruited 35 naive participants, among whom 28 (including 17 females; *M*_age_ = 26.9, s.d._age_ = 6.25) met the minimum RHI threshold for participation (for a description of this assessment, see electronic supplementary material, appendix A and B). This inclusion criterion was crucial, as participants needed to experience the illusion in order to perform the task meaningfully. Previous studies have reported that around 20–25% of individuals do not experience the illusion [53]. This criterion is consistent with standard practice in studies that focus primarily on the illusion itself [27,54–58]. Finally, no distinction was made between left- and right-handed participants given that dominant and non-dominant hands produce similar RHIs [59].

The study received ethical approval from the Ethical Review Authority, and all participants provided written informed consent in accordance with the Declaration of Helsinki. They received compensation of 300 SEK for their participation, and the study took approximately 2 h to complete.

### 2.2. Experimental set-up

Participants rested their right hand, palm facing down, on a flat support surface positioned beneath a wooden table, approximately 30 cm from their body midline. On top of this table, which was elevated 15 cm above the real hand and tilted upward at a 30° angle, two identical rubber hands were arranged side by side in anatomically natural positions within peripersonal space. Each rubber hand was placed at an equal distance of 5 cm to the left or right of the participant’s real hand (figure 1A). This arrangement made it possible to induce the RHI on both rubber hands at the same time [30–32,60,61]. A small white fixation point was located between the two rubber hands, and both a chin rest and an elbow rest (Ergorest Oy^®^, Finland) were used to stabilize the participant’s head and keep the right arm comfortably relaxed throughout the session.

**Figure 1.**
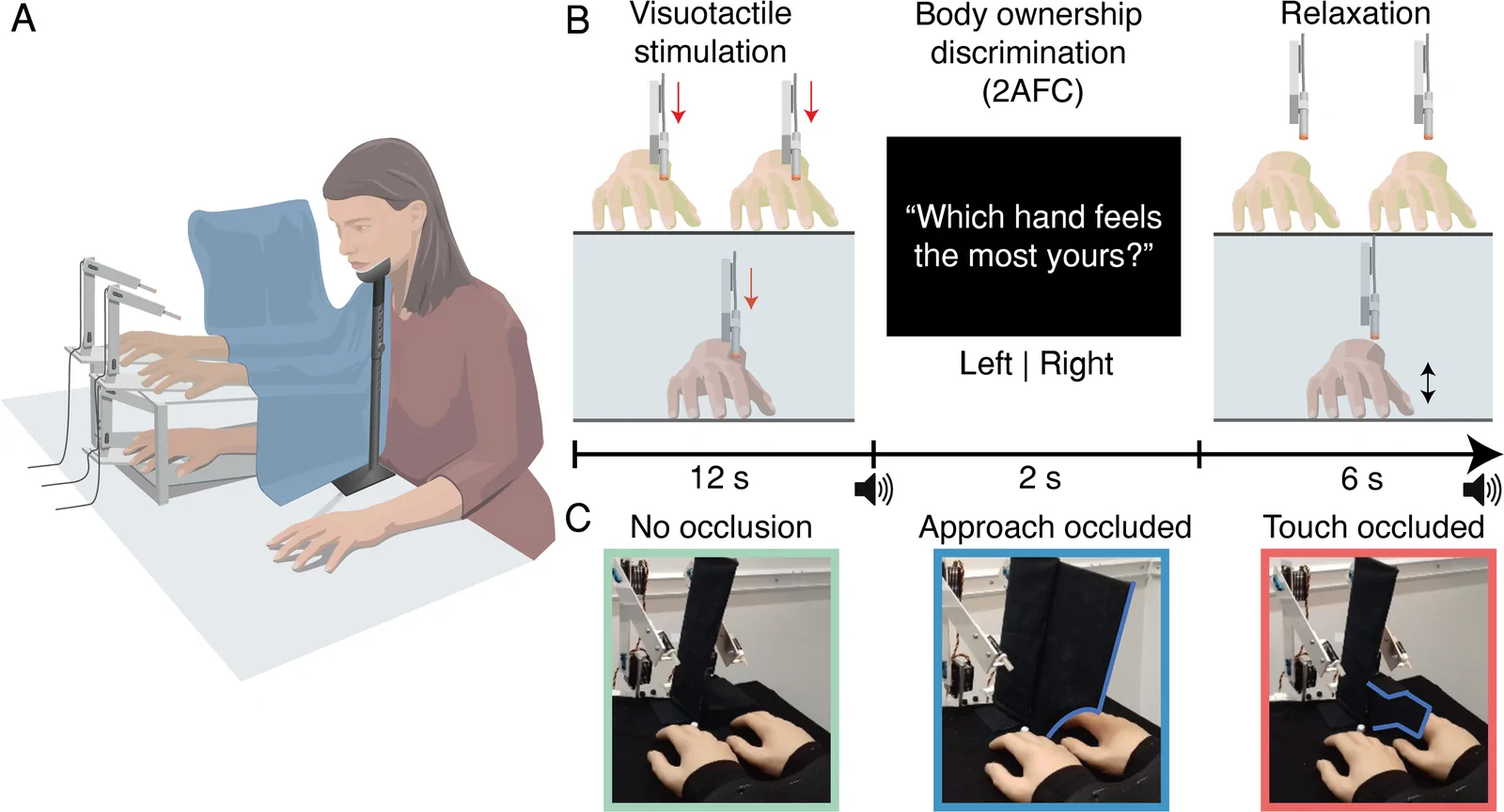
(A) Experimental set-up. Two robotic arms apply touches to two rubber hands placed on top of the platform, while a third robotic arm applies touches to the participant’s real hand, which is hidden beneath it on a lower platform. A white fixation dot is located halfway between the two rubber hands. (B) Trial schematics. The robotic arms tap the rubber hands and real hand with different degrees of asynchrony between the rubber hands; crucially, one rubber hand is always synchronously tapped with the real hand, which is the condition known to produce the strongest RHI. Next, an auditory cue informs participants that they must verbally respond which hand felt most like their own (left or right). Another auditory cue signals the start of the next trial. (C) Visibility conditions. A frame of firm cardboard was placed 5 cm to the left of the right rubber hand. There were three visibility conditions: No occlusion condition—both the touch and the approaching movements of the right robotic arm were visible to the participant; Approach occluded condition—a small screen occluded the participant’s view of the right robotic arm’s approaching movements but not the touch on the rubber hand; and Touch occluded condition—a small screen occluded the participant’s view of the right robotic arm’s touch on the rubber hand but not its approaching movements.

Tactile stimulation was delivered to the index fingers of both rubber hands and the participant’s real hand using three robotic arms (figure 1B). Each robotic arm consisted of two metal segments, each 17 cm long and 3 cm wide, connected to a metal base measuring 10 cm by 20 cm. The movement of the arm joints, including the connection between the two metal segments and between the proximal and supporting parts, was controlled by HS-7950TH UltraTorque servomotors containing 7.2 V optimized coreless engines (Hitec Multiplex^®^, USA). At the end of the distal segment, a ring held a plastic tube with a 7 mm diameter, which delivered the tactile taps. E3X-HD41 fibre sensors (OMRON^®^, The Netherlands) detected the exact moment of contact by measuring the time it took for a red laser beam to reflect off the surface of the hands. These sensors ensured that the intended and actual degrees of asynchrony closely matched. To prevent auditory distractions from the robotic arms, participants wore earphones and listened to white noise during the entire experiment. The noise level was individually adjusted so that it masked the sounds of the robotic movements while remaining comfortable to listen to.

A frame of firm cardboard (8.5 cm × 24 cm) was placed 5 cm to the left of the right rubber hand. This frame did not block participant’s view of the rubber hands or of the robotic arms tapping them; it served to hold the two different screens that occluded the robotic arms’ movements (see below).

### 2.3. Procedure

Participants were instructed to keep their eyes on a visual marker while the robotic arms repeatedly tapped their index fingers. Each trial consisted of six taps delivered within a 12 s period at regular 0.5 Hz intervals and a constant speed of 9 cm s^−1^. The taps were applied to three specific areas on the finger: the second knuckle, the third knuckle and the space between them. The order of stimulation was randomized but identical for both hands to maintain consistency and to avoid any skin irritation. After the sequence of taps, a 500 ms single auditory tone signalled participants to perform a 2AFC task, during which they reported which of the two rubber hands felt most like their own. They were then asked to relax and gently move their fingers to dispel any lingering illusion and to prevent muscle stiffness before the next trial. The beginning of each new trial was indicated by another auditory cue (figure 1B). Previous studies have shown that the RHI can be induced within about 10 s of stimulation [28,54,55], which we confirmed during preliminary testing.

Three degrees of visuotactile asynchrony were applied whereby either the left or right rubber hand was subjected to asynchronous taps compared to the real hand (50, 100 or 200 ms; the 0 ms trials were excluded from SDT because hits/FA are undefined at perfect symmetry). In addition, three different visibility conditions were applied (no occlusion, approach occluded and touch occluded). The approach-occluded condition involved obstructing participants’ view of the first two-thirds of the robotic arm’s approaching movement, i.e. two-thirds (approx. 0.66 s) of the full movement. The movement of the robotic arm in all conditions lasted 1 s (±8 ms). In contrast, the touch-occluded condition concealed their view of the final third of the robotic arm’s movement, including the actual touch, which endured for approximately 0.34 s. Notably, these occlusion conditions were exclusively applied to the right robotic arm, with the left robotic arm always remaining fully visible. The distinctions among these three conditions pertained to the presence or absence of a small occlusion screen and the screen’s shape, size and positioning concerning the right robot’s movement trajectory. Specifically, the no-occlusion condition entailed no screen at all, the approach-occluded condition featured a larger screen measuring 19.5 cm × 24 cm, designed to block the initial ≈0.66 s of the robotic arm’s movement, thereby preventing participants from forming tactile predictions based on visual information during this phase. Meanwhile, the touch-occluded condition utilized a smaller screen measuring 17 cm × 5.5 cm to hide the final ≈0.34 s of the robotic arm’s movement, specifically the tactile interaction itself (figure 1C). The experiment comprised six blocks (two per visibility condition), 42 trials per block, with each asynchrony level repeated six times in randomized order, with a 6 s intertrial interval. Before each block, screen placement and visibility were verified as in Chancel *et al.* [27].

### 2.4. Analysis

To quantify performance in the 2AFC task, we analysed the data within an SDT framework [30,35]. For each participant, we computed SDT measures separately for every combination of visuotactile asynchrony and visibility condition. We followed the convention of treating trials in which the *right* rubber hand was synchronous with the real hand as ‘signal-present’ trials and trials in which the synchronous stimulation occurred on the *left* as ‘signal-absent’ trials [30,31]. The proportion of ‘right-hand’ responses on signal-present trials was denoted $P_{\text{hit}}$, and the proportion of ‘right-hand’ responses on signal-absent trials was denoted $P_{\text{FA}}$. Body-ownership sensitivity was expressed as *d*′ using the standard 2AFC formula: $d_{\text{ownership}}^{\prime }=\left(\frac{1}{\sqrt{2}}\right)\left(Z\left(P_{hit}\right)-Z\left(P_{FA}\right)\right)$, where the *Z*-scores of $P_{\text{hit}}$ and $P_{\text{FA}}$ were calculated. Higher *d*′ values indicate better discrimination between synchronous and asynchronous visuotactile stimulation, independently of any tendency to favour one rubber hand over the other. Bias towards one of the two rubber hands was characterized by the decision criterion index: $C_{\text{rubber hand}}=-\left(\frac{1}{2}\right)\left(Z\left(P_{hit}\right)+Z\left(P_{FA}\right)\right)$. Positive values of C reflect a greater tendency to choose the left rubber hand, whereas negative values indicate a bias towards the right hand. Thus, C provides a summary of both the direction and strength of participants’ ownership judgements across conditions.

For the statistical analysis, we used repeated-measures analysis of variance (ANOVA) with the degree of visuotactile asynchrony (50, 100 and 200 ms) and the visibility condition (no occlusion, approach-occluded and touch-occluded) as factors to examine how these two manipulations influenced both body ownership sensitivity and perceptual bias. When Mauchly’s test revealed a violation of the sphericity assumption, we applied the Greenhouse–Geisser adjustment to correct the degrees of freedom. Significant interactions (*p* < 0.05) were followed up with *post hoc* pairwise comparisons using Holm–Bonferroni correction, calculated from the pooled variance of the ANOVA model and based on estimated marginal means. Because the absence of a significant result in a frequentist framework does not necessarily indicate the absence of an effect, we also complemented our analysis with a Bayesian repeated-measures ANOVA [62,63]. This approach allowed us to quantify the relative evidence for alternative hypotheses compared with the null model. Bayes factors were computed using a Cauchy prior distribution centred at zero with a scale parameter of 0.707, which represents the standard default choice when prior information is unavailable [62–64]. All statistical analyses were conducted in MATLAB 2022b (MathWorks, Inc.) and JASP [65], and the results were cross-checked in R to ensure consistency.

## 3. Results

### 3.1. The effect of visual occlusion on body ownership sensitivity

Body ownership sensitivity scores (*d*′) were analysed using a repeated-measures ANOVA. The analysis revealed a strong main effect of asynchrony $\left(F_{\left(2,54\right)}=220.41,\;p<0.001,\;\eta p^{2}=0.891\right)$, indicating that variations in asynchrony significantly influenced sensitivity. In contrast, neither the visibility factor $\left(F_{\left(2,54\right)}=1.88,\;p=0.163,\;\eta p^{2}=0.065\right)$ nor the interaction between visibility and asynchrony $\left(F_{\left(4,108\right)}=1.15,\;p=0.338,\;\eta p^{2}=0.041\right)$ reached statistical significance (figure 2A,B; also see electronic supplementary material, appendix C). *Post hoc* pairwise tests confirmed that sensitivity did not differ between any of the visibility conditions: approach-occluded versus no-occlusion (t(27)=1.17, p=0.494, d=0.174), approach-occluded versus touch-occluded (t(27)=0.751, p=0.494, d=0.112) or touch-occluded versus no-occlusion (t(27)=1.922, p=0.180, d=0.286). Bayesian analysis further supported these findings, providing strong evidence for the null model regarding visibility $\left({\text{BF}}_{01}=3.434\right)$ and decisive evidence for the alternative model involving asynchrony $\left({\text{BF}}_{10}>100\right)$, confirming that visuotactile asynchrony robustly modulates ownership sensitivity (figure 2C,D). Taken together, these results indicate that body ownership sensitivity to visuotactile synchrony remains stable even when visual information about the robotic arm’s motion or contact is unavailable.

**Figure 2.**
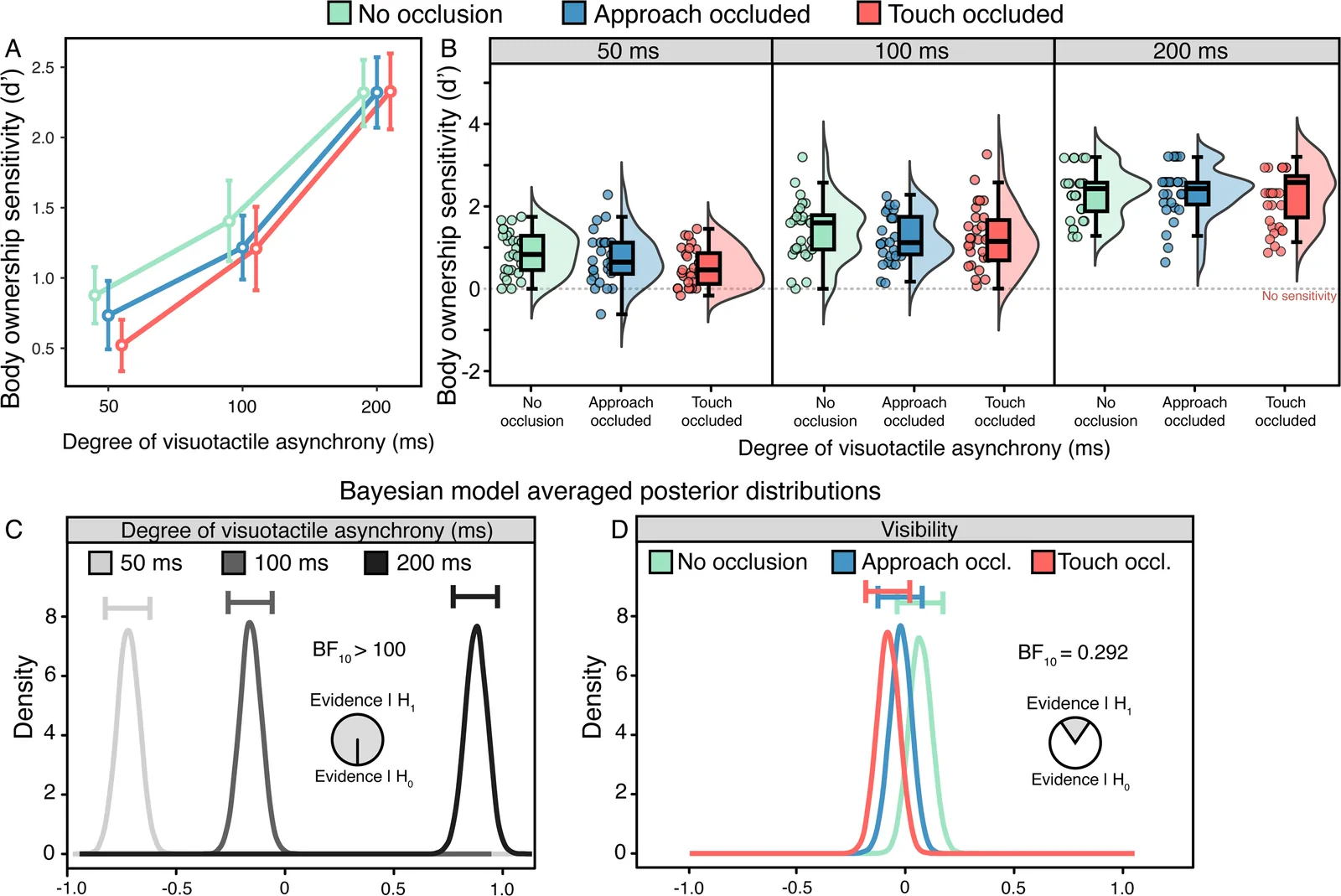
Body ownership sensitivity results. (A,B) Body ownership sensitivity increased with increasing degree of asynchrony of tactile tapping between rubber hands, but it did not significantly differ between visibility conditions. (C) Posterior distributions of the effects of degree of visuotactile asynchrony demonstrate extreme support for the alternative hypothesis model, i.e. that body ownership sensitivity differs between different degrees of visuotactile stimulation asynchrony. (D) Posterior distributions of the effects of visibility (occlusion) demonstrate strong support for the null hypothesis model, i.e. that body ownership sensitivity does not differ between visibility conditions. Error bars denote 95% confidence intervals (A) and 95% credible intervals (C,D).

### 3.2. The effect of visual occlusion on perceptual bias

Overall, we observed a perceptual bias favouring the left rubber hand, which may reflect that this model hand was positioned closer to the body midline, a factor that has influenced the illusion in specific experimental configurations [28,30,66]. However, this preference decreased as the degree of visuotactile asynchrony increased (figure 3A). This effect was supported by a series of (uncorrected) one-sample *t*-tests against zero, where positive scores indicated a bias towards the left rubber hand and negative scores indicated a bias towards the right. All comparisons were significant (*p* < 0.037), except for the no-occlusion condition at 200 ms of asynchrony (*p* = 0.566) and the touch-occluded condition at 100 ms (*p* = 0.169) and 200 ms of asynchrony (*p* = 0.669).

**Figure 3.**
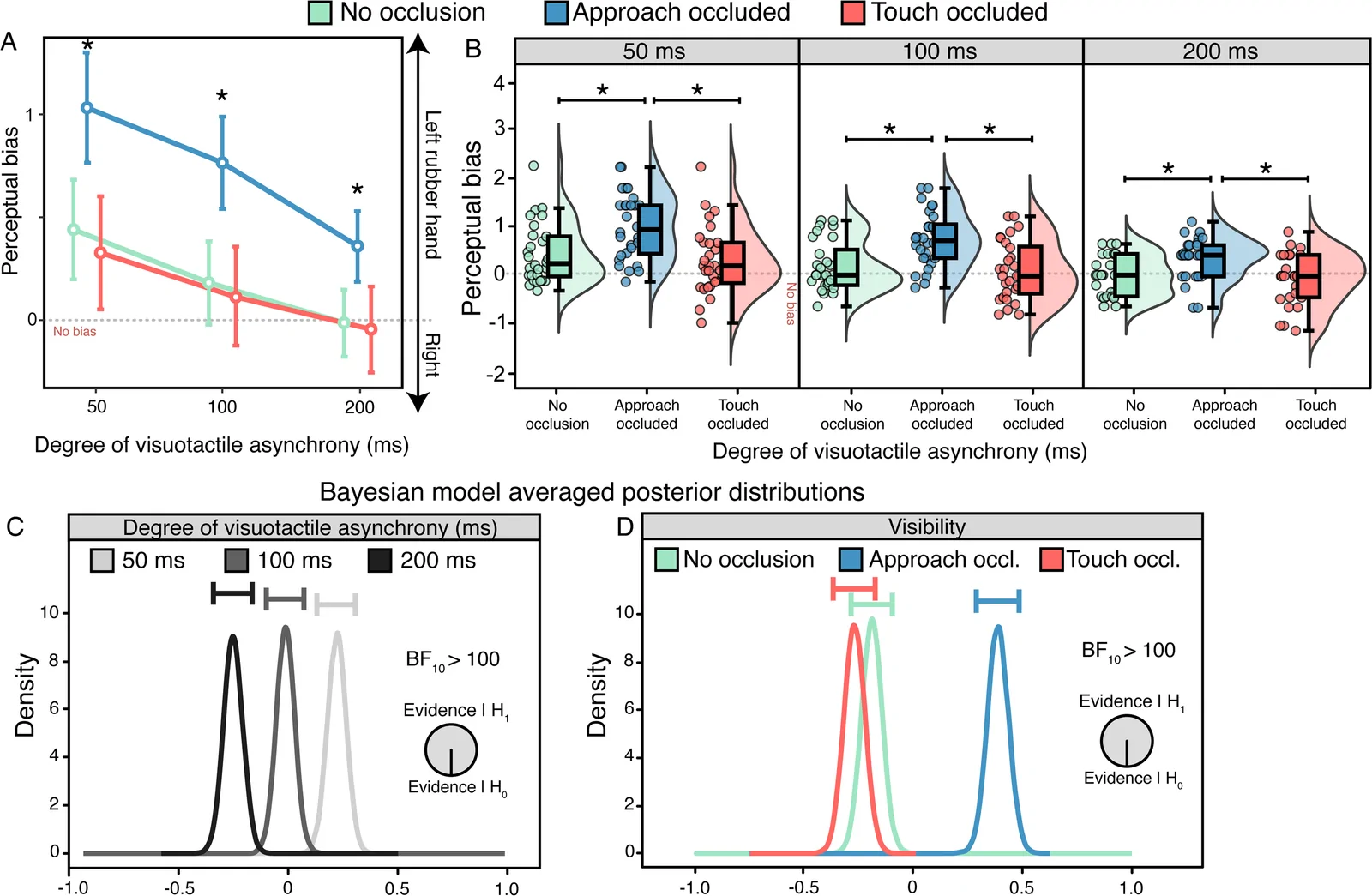
Perceptual bias results. (A,B) Perceptual bias decreased with increasing visuotactile asynchrony between the rubber and real hands and showed a significant preference for the left rubber hand in the approach-occluded condition, where only the right rubber hand’s visibility was manipulated. (C) Posterior distributions of the effects of degree of visuotactile asynchrony demonstrate extreme support for the alternative hypothesis model, i.e. that perceptual bias differs between different degrees of visuotactile stimulation asynchrony. (D) Posterior distributions of the effects of visibility (occlusion) demonstrate extreme support for the alternative hypothesis model, i.e. that perceptual bias differs between visibility conditions. Error bars denote 95% confidence intervals (A) and 95% credible intervals (C,D).

The analysis revealed strong main effects of both visuotactile asynchrony and visibility on perceptual bias. The effect of asynchrony was significant $\left(F_{\left(1.35,36.40\right)}=24.69,\;p<0.001,\;\eta p^{2}=0.478\right)$, as was the effect of visibility $\left(F_{\left(2,54\right)}=44.20,\;p<0.001,\;\eta p^{2}=0.621\right)$. However, the interaction between these factors was not significant $\left(F_{\left(4,108\right)}=1.65,\;p=0.166,\;\eta p^{2}=0.058\right)$ (figure 3A,B; also see electronic supplementary material, appendix D). *Post hoc* analyses showed clear differences between the approach-occluded and no-occlusion conditions (t(27)=8.6, p<0.001, d=1.430), as well as between the approach-occluded and touch-occluded conditions (t(27)=7.46, p<0.001, d=1.628). There was no significant difference between the no-occlusion and touch-occluded conditions (t(27)=1.11, p=0.277, d=0.198). These outcomes are consistent with the PSE findings previously reported by Chancel *et al.* [27]. Bayesian analysis provided very strong evidence that both visibility and visuotactile asynchrony affected perceptual bias (BF_10_ > 100 for both comparisons; figure 3C,D). Overall, the results show that perceptual bias, reflected in a greater tendency to attribute ownership to the left rubber hand, increased when participants were unable to form tactile predictions from visual information in the approach-occluded condition.

## 4. Discussion

The sense of body ownership depends primarily, but not exclusively, on the integration of visual and tactile signals. An important unresolved question concerns how predictions about touch, derived from visual cues of an object approaching one’s body, influence this experience. Previous work suggested that occluding visual information necessary for predicting touch, but not the sight of the touch itself, reduces body ownership [27]. In the present study, we reanalysed the datasets from Chancel *et al.* [27] using an SDT framework to determine whether disrupting visually driven tactile predictions alters the sensitivity of body ownership judgements to visuotactile synchrony or the perceptual bias favouring one rubber hand over the other.

Our main findings are (i) that disrupting visually driven tactile predictions by occluding visual feedback from the first part of the probe’s approach towards the rubber hand does not impair the sensitivity of body ownership to visuotactile signal processing and (ii) that disrupting visuotactile integration by occluding the visual feedback of the actual visuotactile touch event as it occurs does not impair body ownership sensitivity when sufficient visual information is available from the approach movement to infer the motion trajectory. Thus, as long as touch on the rubber hand can be either predicted or seen, visuotactile integration processing is preserved. Taken together, this pattern suggests that effective visuotactile integration supporting the sense of ownership can operate when the visual system either predicts an upcoming touch or directly perceives contact with the rubber hand. However, we observed a shift in perceptual bias, specifically when tactile predictions were disrupted, and not when visual feedback of touch was occluded. This suggests that visually driven tactile predictions shape body ownership judgements through a bias mechanism beyond the direct integration of bottom-up visual and tactile signals.

One possible explanation for this bias effect is the absence of visual stimulation within the peripersonal space surrounding the hand when the approaching probe is occluded. Seeing an object move towards the hand normally activates multisensory neurons in parietal and premotor areas that encode near-hand space in visuotactile coordinates [6,19,67,68]. These neurons contribute to representing the body’s immediate surroundings and are thought to facilitate the integration of stimuli that occur close to the hand. When this visual stimulation in peripersonal space is absent, the spatial coupling between the visual and tactile modalities may be weakened, leading to a reduction in perceptual bias for the corresponding rubber hand.

Alternatively, this bias effect may reflect a higher-order contextual influence of tactile predictions on the integration of visual and proprioceptive signals in the RHI, which is known to depend on the spatial congruence between seen hand location and felt hand posture [30,55,69,70]. Although visual hand location and proprioceptive posture were constant across conditions, disrupting visually driven tactile predictions may reduce the effective contribution of visual hand information relative to proprioceptive signals in ownership judgements, thereby biasing responses. Another possibility is that visually driven tactile predictions influence body ownership through top-down inference mechanisms described in predictive-coding frameworks [71]. When the sight of the approaching probe allows the brain to predict the timing and location of an impending touch, the subsequent tactile input can be matched against this prediction. A close match minimizes prediction error and reinforces the percept that the seen and felt events arise from a common cause, namely, one’s own body. Conversely, when visual information about the approaching probe is occluded, tactile inputs arrive as less predictable, producing larger prediction errors and a weaker inference of ownership for that hand. Within this framework, our observed shift in perceptual bias when tactile predictions were disrupted can be interpreted as a change in the prior belief that the seen limb belongs to oneself: reduced predictive precision weakens the top-down expectation of self-related touch, thereby biasing ownership judgements away from the occluded hand [72,73].

As said, our analysis revealed a specific modulation of the illusion’s perceptual bias when tactile predictions were disrupted, thus replicating Chancel *et al.*’s PSE findings [27]. Participants for whom visual feedback was occluded during the initial two-thirds of the robot’s approach towards the right rubber hand exhibited a significantly greater perceptual bias in favour of the left rubber hand compared to the other two conditions, where only touch was visually occluded or nothing was occluded. Across all conditions, we observed that participants’ perceptual bias was most pronounced with lower degrees of visuotactile asynchrony when multisensory signal differences were most uncertain. However, as visuotactile asynchrony increased and body ownership sensitivity heightened, the perceptual bias favouring the rubber hand closer to the body decreased, which is a pattern consistent with our previous studies [30,31]. Even when tactile predictions were weakened by restricting visual feedback of the approaching object, the pattern persisted, accompanied by a stronger perceptual bias. This may suggest that occluding the robot arm’s approaching movements and, consequently, limiting visual information for tactile predictions hindered participants’ feeling of ownership with that specific rubber hand, which is consistent with the view that the visually driven tactile predictions shape body ownership judgements through a perceptual bias mechanism.

Our results indicate that the *core* multisensory mechanism underlying the RHI is preserved even when predictive visual information is disrupted. Across conditions, changes in visuotactile timing produced large and reliable differences in our SDT sensitivity measure, replicating previous demonstrations that body ownership judgements are highly sensitive to visuotactile asynchrony [28,30,31]. Crucially, this sensitivity did not differ between the approach-occluded and no-occlusion conditions, despite the clear effect of occlusion on perceptual bias. This pattern is consistent with earlier work identifying visuotactile synchrony as a primary driver of the illusion [28,51,74] and supports the interpretation of SDT-derived sensitivity as a bias-independent index of multisensory integration. At the same time, the present findings reinforce the idea that bottom-up visuotactile correlations and top-down, prediction-related processes both shape body ownership, but do so through separable contributions to sensitivity and bias.

The SDT analysis presented here complements and extends the psychophysical approach used by Chancel *et al.* [27]. In their study, condition-dependent shifts in the PSE indicated that the RHI was reduced when visually driven tactile predictions were disrupted. Our SDT analysis refines this interpretation by showing that this effect reflects a reduction in perceptual bias rather than a change in body ownership sensitivity to visuotactile asynchrony, thereby deepening our understanding of how multisensory predictions contribute to body ownership. Together, these findings are consistent with those of Chancel *et al.* [27] and demonstrate that removing predictive visual cues alters perceptual bias in body ownership, a dissociation that is clearly revealed by the SDT framework. Moreover, Chancel *et al.* [27] reported the lowest proportion of ownership judgements when only the final visuotactile contact was visible, which could be taken to suggest a dominant role of tactile predictions in the illusion. The present SDT results refine this interpretation by showing that body ownership sensitivity to visuotactile synchrony remains stable across conditions, indicating that the integration of bottom-up visual and somatosensory signals is preserved despite the disruption of visually driven predictions.

An interesting avenue for future research would be to investigate the neural mechanisms underlying the visually driven tactile prediction effect on body ownership. Previous functional magnetic resonance imaging (fMRI) studies have implicated regions such as the ventral premotor cortex, posterior parietal cortex, lateral occipital–temporal cortex and cerebellum in the integration of visual, tactile and proprioceptive information, particularly within peripersonal space, in the RHI [54,67,68,75–79]. However, little is known about how these regions implement multisensory predictions that build up prior to the moment when the seen object touches the rubber and real hands, and how such predictive neural responses relate to the multisensory integration-related activity that occurs after each visuotactile contact [77,80,81]. The present robotic RHI occlusion paradigm, combined with the SDT analytic approach, could be applied in future fMRI, electroencephalogram and magnetoencephalogram experiments to address these questions and to link neural activity in the RHI to both body ownership sensitivity and perceptual bias, thereby providing deeper insight into the underlying neural mechanisms.

Future studies could also further explore how other sensory inputs, and predictions about them, influence multisensory processing and perceptual bias in body ownership illusions. For example, proprioception-driven predictions could be examined in the non-visual RHI driven by passive self-touch [76] or efference copy-driven predictions in the RHI elicited by active finger movements [82]. Since body ownership is the result of the accumulation of multisensory evidence that builds up over repeated visuotactile stimulations [32,83,84], it would also be interesting to clarify whether this buildup of multisensory evidence is based solely on sensory feedback from the touch events or whether visually driven tactile predictions also contribute to this process.

A limitation of the present study concerns the interpretation of the observed bias associated with disrupted visually driven predictions. Although our SDT analysis suggests that this bias reflects a perceptual rather than a cognitive effect, it remains possible that attentional factors contributed to the result. Specifically, when the initial two-thirds of the robot arm’s movement was occluded, the approaching stimulus may have been less visually salient or attention-grabbing, thereby introducing a cognitive bias towards the non-occluded arm. Such an attentional asymmetry could influence response tendencies independently of perceptual changes in body ownership. Future studies could address this possibility by incorporating measures of spatial attention or by using control conditions where attentional capture is explicitly manipulated. More generally, disentangling perceptual and cognitive contributions to bias effects in body ownership paradigms remains an important challenge for understanding the mechanisms underlying predictive multisensory processing.

Finally, in the current experiment, the motion trajectory behind the occluder was always presented at a constant and relatively short distance from the rubber hand, and with a fixed duration (333 ms). These parameters are known to provide optimal conditions for amodal motion completion and apparent motion perception [85–87]. Neurons in motion-sensitive regions such as MT/V5 and parietal cortex can represent inferred motion trajectories across occlusion [88], supporting the continuity of perceived movement. Maintaining short and consistent trajectories likely enhanced perceptual continuity and visuotactile predictability, which facilitate motion-based perception and body ownership [89,90]. Because tactile predictions probably depend on such predictive visual motion processing, future experiments could manipulate the predictability of visual feedback. This could be done by varying speed (constant and predictable versus random and unpredictable), distance (long, starting outside peripersonal space versus short, as in the current experiment) or direction (towards the hand versus towards a point beside it). Such manipulations would allow testing how the precision and strength of inferred contact events behind the occluder influence tactile predictions and, in turn, the experience of body ownership.

In conclusion, our findings suggest that the integration of visual and tactile signals underlying body ownership remains effective as long as visual information is available either to generate tactile predictions or to provide direct evidence of touch. However, occluding visual feedback that enables such tactile predictions alters the perceptual bias underlying the RHI. These results demonstrate that predictive visual mechanisms make a distinct and measurable contribution to body-ownership perception beyond the integration of concurrent sensory inputs.

## Supplementary Material



## Data Availability

The data are available from the OSF repository at: https://osf.io/tcwmu/. Electronic supplementary material is available online [91].

## Supplement

Supplementary material is available online at https://doi.org/10.6084/m9.figshare.c.8381675.
